# Single probes and resonant four-wave-mixing enabling novel correlative light electron microscopy workflow

**DOI:** 10.1038/s41377-023-01154-x

**Published:** 2023-05-08

**Authors:** Long Chen, Chao He

**Affiliations:** 1 Division of Structural Biology, Wellcome Trust Centre for Human Genetics, University of Oxford, Oxford, OX3 7BN UK; 2 Department of Engineering Science, University of Oxford, Oxford, OX1 3PJ UK

**Keywords:** Optics and photonics, Optical techniques

## Abstract

Correlative light electron microscopy prefers single probes with stable performance in both optical and electron microscopy. Now researchers have shown how to harness gold nanoparticles featuring exceptional photostability and four-wave-mixing nonlinearity to realize a new correlation imaging approach.

Advances in correlative light-electron microscopy (CLEM) have enabled various novel explorations in life science including neuroscience, microbiology, cellular and molecular biology via complementing functional and ultrastructural information. Development is going on at an unprecedented speed^1,2^. CLEM is a combination of both electron microscopes (EM) and their optical counterparts (fluorescence microscopes in general), in which robust probes with stable performance are highly demanded for both microscopy techniques in order to accurately identify and locate specific bio-structures. Previous research has revealed that both dual and single probes can work for CLEM—such as fluorescent moiety in conjunction with a gold nanoparticle (AuNP), single fluorophores and quantum dots. However, these candidates suffer from various issues for biological applications, including quenching, photobleaching, cytotoxicity and intermittent emission^3–5^. Using AuNPs as single probes with their nonlinearity characterized by four-wave mixing (FWM) microscopy is an exciting prospect to overcome the aforementioned problems^6^.

In a recently published paper^7^, Iestyn Pope and co-authors, in the laboratories headed by Prof. Paola Borri at Cardiff University and Prof. Paul Verkade at the University of Bristol, ingeniously combine the superiorities of AuNPs (photostable) and FWM (completely free from background), facilitating CLEM using small AuNPs as single probes. AuNPs are visible under both modalities, hence high-accuracy image correlation can be achieved without the need for additional fiducial markers. For FWM, a multiphoton technique is employed to excite the nonlinearity of AuNPs, via a triply resonant scheme, and detect individual AuNPs within cell sections, free from any background^6^. The epidermal growth factor (EGF) protein coupled to a AuNP was internalized into HeLa cells for a demonstration in the work. The cell sample preparation was performed with high-pressure freezing and freeze substitution without utilizing heavy metal stains, and 300 nm thin resin sections were cut for analysis and were snapshotted by FWM and transmission EM (TEM). With this CLEM tool, the authors demonstrate a correlation accuracy <60 nm over an area larger than 10 µm, using 5 nm and 10 nm radius nanoparticles, which can be brought towards <40 nm and even <10 nm when removing all systematic errors i.e. reaching the localization precision shot-noise limit. A schematic workflow of FWM-CLEM is shown in Fig. 1.

**Fig. 1:**
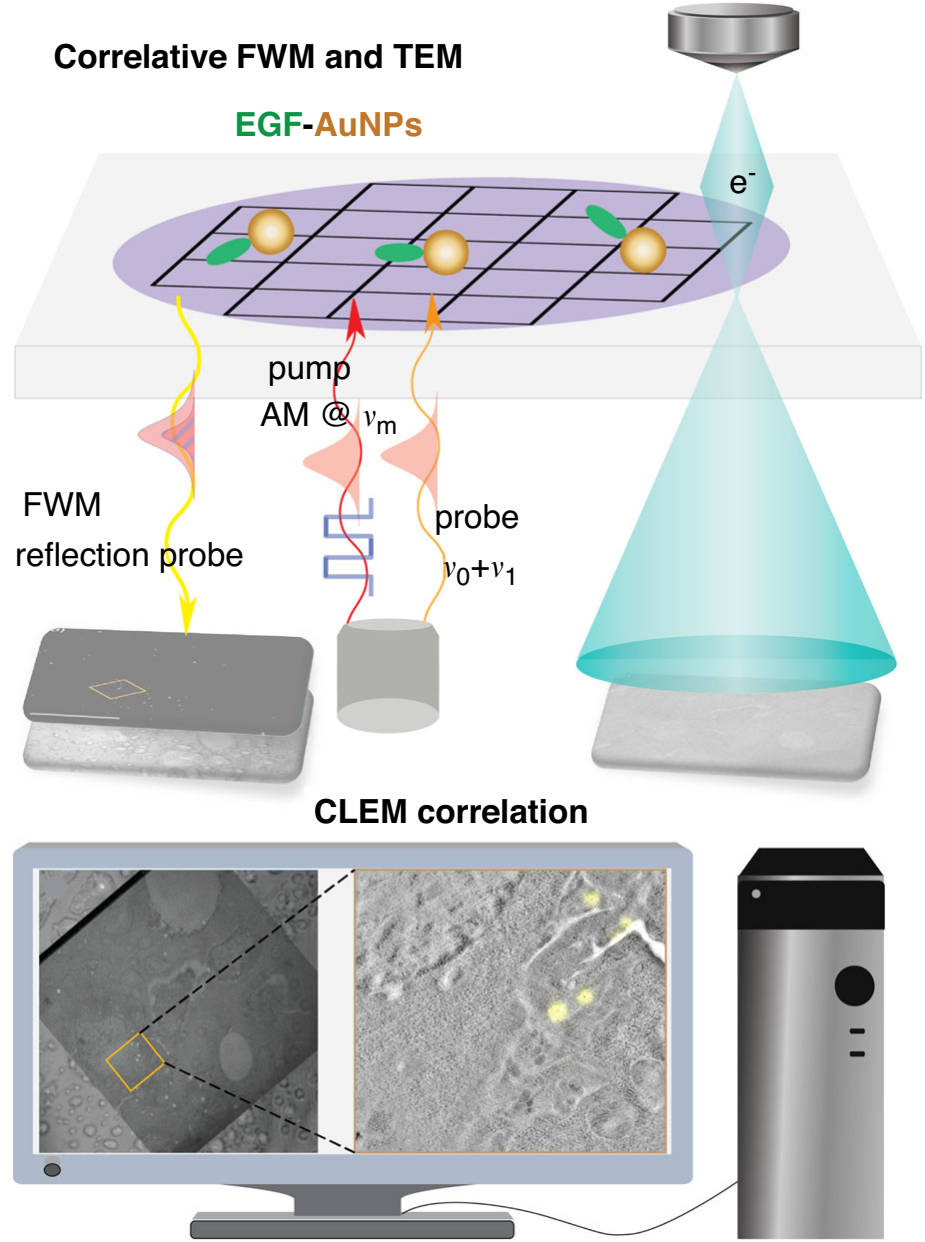
A schematic correlative FWM and TEM workflow. EGF-conjugated AuNPs in cells were detected by FWM and TEM, allowing for high-accuracy image correlation across modalities without additional fiducial markers. AM amplitude modulation

It is clear that the combination of utilizing AuNPs and the FWM technique facilitates this breakthrough. Compared with the majority of other probes, a AuNP enables fast and long-time imaging and is not prone to photobleaching. Notably, FWM is compatible with dynamic imaging, and could be used for single molecule tracking (such as the spatio-temporal observation of the fate of individual virions in live cells)^8^. In addition, AuNPs can be genetically encoded in cells as EM-visible markers and designed to target specific biomolecules^9^—which potentially fills the ‘specificity’ for FWM optical microscopy. With FWM-CLEM tagged by AuNPs, events of interest in the cellular ultrastructure can be pin-pointed and magnified to reveal ultrastructure details.

The team discusses the possibilities and potential outreaching of this work, including harnessing the polarization response of the nanomaterials as well as the FWM nonlinearity^6^. These phenomena are of great potential to benefit not only the systematic performance of FWM microscopes such as for optical resolution^10^, but also further reveal the thermal and mechanical properties of the local environment surrounding the probes^11^. Furthermore, polarization resolved FWM can map the polarization anisotropy associated with the shape information of nanoparticles, which can enable access to additional structural details of the labeled biomedical targets^6^. Building upon these advances in recent FWM-CLEM, there is still work to be done. The current FWM implementation is not fast enough in terms of imaging speed, but this is likely to be improved via engineering solutions, e.g., adopting fast galvo mirrors. There are also prospects for further extension, e.g., FWM development in cryogenic states. Through this view, cryo-FWM-CLEM might be conducted to improve the efficiency of cryo-CLEM and advance in-situ cellular biology research. The present work highlights the inspiring prospects in FWM microscopy and FWM-CLEM, which paves the way for next-generation single probe CLEM workflow.
